# Halide Double Perovskite Nanocrystals Doped with Rare‐Earth Ions for Multifunctional Applications

**DOI:** 10.1002/advs.202207571

**Published:** 2023-04-28

**Authors:** Xin Li, Dingdi Wang, Yuan Zhong, Feng Jiang, Deqiang Zhao, Siqi Sun, Po Lu, Min Lu, Zhenyu Wang, Zhennan Wu, Yanbo Gao, Yu Zhang, William W. Yu, Xue Bai

**Affiliations:** ^1^ State Key Laboratory of Integrated Optoelectronics and College of Electronic Science and Engineering Jilin University Changchun 130012 China; ^2^ Department of Materials and Environmental Chemistry Stockholm University Stockholm SE10691 Sweden; ^3^ School of Chemistry and Chemical Engineering Shandong University Jinan 250100 China

**Keywords:** halide double perovskite nanocrystals, lead‐free, rare‐earth ions, anti‐counterfeiting, optical thermometry, white‐light‐emitting diodes

## Abstract

Most lead‐free halide double perovskite materials display low photoluminescence quantum yield (PLQY) due to the indirect bandgap or forbidden transition. Doping is an effective strategy to tailor the optical properties of materials. Herein, efficient blue‐emitting Sb^3+^‐doped Cs_2_NaInCl_6_ nanocrystals (NCs) are selected as host, rare‐earth (RE) ions (Sm^3+^, Eu^3+^, Tb^3+^, and Dy^3+^) are incorporated into the host, and excellent PLQY of 80.1% is obtained. Femtosecond transient absorption measurement found that RE ions not only served as the activator ions but also filled the deep vacancy defects. Anti‐counterfeiting, optical thermometry, and white‐light‐emitting diodes (WLEDs) are exhibited using these RE ions‐doped halide double perovskite NCs. For the optical thermometry based on Sm^3+^‐doped Cs_2_NaInCl_6_:Sb^3+^ NCs, the maximum relative sensitivity is 0.753% K^−1^, which is higher than those of most temperature‐sensing materials. Moreover, the WLED fabricated by Sm^3+^‐doped Cs_2_NaInCl_6_:Sb^3+^ NCs@PMMA displays CIE color coordinates of (0.30, 0.28), a luminous efficiency of 37.5 lm W^−1^, a CCT of 8035 K, and a CRI over 80, which indicate that Sm^3+^‐doped Cs_2_NaInCl_6_:Sb^3+^ NCs are promising single‐component white‐light‐emitting phosphors for next‐generation lighting and display technologies.

## Graphical abstract

Rare‐earth ions are introduced into the Sb^3+^‐doped Cs_2_NaInCl_6_ nanocrystals (NCs), which endow double perovskite NCs with outstanding and tunable emissions, and a photoluminescence quantum yield of 80.1% is achieved in Tb^3+^‐doped Cs_2_NaInCl_6_:Sb^3+^ NCs. Meanwhile, the double functions of Tb^3+^ are investigated by emtosecond transient absorption spectra. Finally, luminescence‐related applications are demonstrated, including anti‐counterfeiting, optical thermometry, and white‐light‐emitting diodes, exhibiting great prospects.

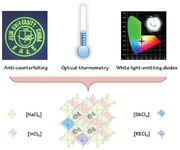

## 1 Introduction

All‐inorganic halide double perovskites with a formula of A_2_B(I)B(III)X_6_ (A = Cs^+^, Rb^+^; B(I) = Na^+^, K^+^, Ag^+^; B(III) = In^3+^, Bi^3+^, Sb^3+^) have attracted wide attention due to the nontoxicity and great stability.^[^ ^1^ ^]^ Generally, halide double perovskites exhibit dim optical characteristics with low photoluminescence quantum yields (PLQYs) because of the indirect bandgap and parity‐forbidden transition.^[^ ^2^ ^]^ Researchers have explored some effective methods to realize the bandgap transformation and parity‐allowed transition via doping or alloying to improve the optical characteristics. Han et al. transformed Cs_2_AgBiCl_6_ from an indirect to a direct bandgap by introducing In^3+^ and obtained the desired results.^[^ ^3^ ^]^ Tang's group broke the parity‐forbidden transition and decreased the defect by introducing Na^+^ and Bi^3+^ into the Cs_2_AgInCl_6_ host, achieving an efficient white emission.^[^ ^4^ ^]^ Typically, halide double perovskites possess low electronic dimensionality and soft lattice, along with strong electron‐phonon coupling, which are beneficial to the self‐trapped exciton (STE) emissions.^[^ ^5^ ^]^ A previous study manifested that the STE emission energy can be expressed as *E*_PL_ = *E*_g_ − *E*_b_ − *E*_st_ − *E*_d_, wherein the *E*_g_, *E*_b_, *E*_st_, and *E*_d_ represent the bandgap energy, exciton binding energy, self‐trapping energy and lattice deformation energy, respectively. In the formula, each part is hard to modulate. Hence, efficient and tunable STE emission is difficult to achieve.^[^ ^6^ ^]^

The incorporation of rare‐earth (RE) ions is an effective strategy to modulate the emission of materials.^[^ ^7^ ^]^ Moreover, their long lifetime, sharp‐band emissions, and excellent optical stability also evoked considerable interests.^[^ ^8^ ^]^ However, most RE ions incorporated luminescent materials display low PLQYs, ascribed to the limitation of the host and the low absorption efficiency of RE ions originating from the parity‐forbidden 4f–4f electronic transition.^[^ ^9^ ^]^ To overcome the shortcomings, it is exceedingly crucial to select an appropriate host. Sb^3+^‐doped Cs_2_NaInCl_6_ nanocrystals (NCs) are regarded as a kind of outstanding host material. First, halide materials possess lower phonon energy than sulfide, oxide, and fluoride host materials, which can decrease nonradiative losses.^[^ ^10^ ^]^ Second, Sb^3+^‐doped Cs_2_NaInCl_6_ NCs show efficient blue light emission, which could be exploited as an antenna for sensitizing RE ions.^[^ ^2b^ ^]^ Finally, Cs_2_NaInCl_6_ possesses more excellent stability than widely reported Cs_2_AgInCl_6_ due to the facile reduction of Ag^+^ to Ag^0^ under light radiation.^[^ ^11^ ^]^ Combining the outstanding properties of halide double perovskite and RE ions, it is worthwhile to investigate the RE ions incorporated Cs_2_NaInCl_6_:Sb^3+^ NCs.

Herein, RE ion‐doped Cs_2_NaInCl_6_:Sb^3+^ NCs were synthesized via a modified hot injection method. The introduction of RE ions (Sm^3+^, Eu^3+^, Tb^3+^, and Dy^3+^) in the perovskite host endowed the halide double perovskite NCs with outstanding and tunable emission proprieties, and the highest PLQY of 80.1% was obtained in Tb^3+^‐doped Cs_2_NaInCl_6_:Sb^3+^ NCs. Moreover, the effects of Tb^3+^ ions in lead‐free halide perovskite NCs are duple, namely filling the vacancy defect and serving as the activator, which were confirmed via femtosecond transient absorption (Fs‐TA) measurement. Furthermore, RE ion‐doped Cs_2_NaInCl_6_:Sb^3+^ NCs were applied to anti‐counterfeiting, optical thermometry, and white‐light‐emitting diode (WLED). The maximum absolute sensitivity and relative sensitivity of Sm^3+^‐doped Cs_2_NaInCl_6_:Sb^3+^ NCs were 0.173 and 0.753% K^−1^ for the optical thermometry. Moreover, the WLED displayed CIE color coordinates of (0.30, 0.28), a luminous efficiency of 37.5 lm W^−1^, a CCT of 8035 K, and a CRI of 82.

## 2 Results and Discussions

**Figure 1:**
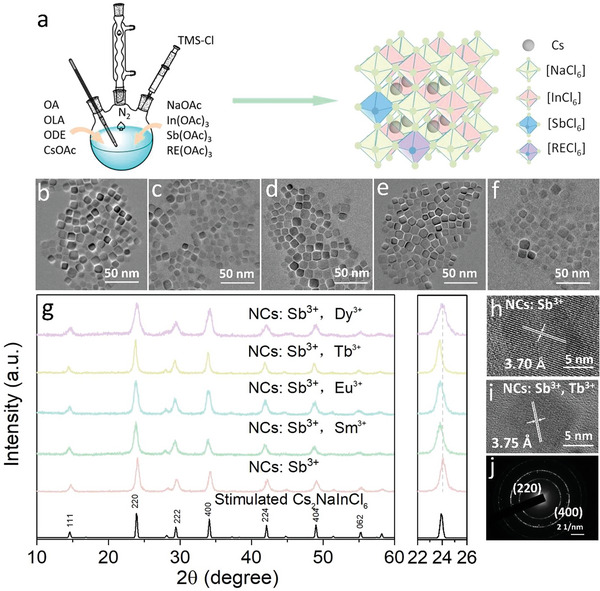
a) Schematic illustration of the colloidal synthesis of RE ions‐doped Cs_2_NaInCl_6_:Sb^3+^ NCs. b‐f) TEM images of pristine Cs_2_NaInCl_6_:Sb^3+^ NCs, and RE ions (Sm^3+^, Eu^3+^, Tb^3+^, Dy^3+^)‐doped Cs_2_NaInCl_6_:Sb^3+^ NCs. g) XRD patterns of pristine Cs_2_NaInCl_6_:Sb^3+^ NCs, RE ions‐doped Cs_2_NaInCl_6_:Sb^3+^ NCs, and the right panel is the magnified XRD patterns at a range of 22°–26°. h,i) HR‐TEM images of Cs_2_NaInCl_6_:Sb^3+^ NCs and Tb^3+^‐doped Cs_2_NaInCl_6_:Sb^3+^ NCs. j) SAED pattern of Tb^3+^‐doped Cs_2_NaInCl_6_:Sb^3+^ NCs.

The synthesis methods of Cs_2_NaInCl_6_:Sb^3+^ NCs and RE ions (Sm^3+^, Eu^3+^, Tb^3+^, Dy^3+^)‐doped Cs_2_NaInCl_6_:Sb^3+^ NCs were developed via modifying the preparation of Cs_2_NaInCl_6_ NCs reported by Han et al. ^[^ ^2a^ ^]^ As shown in **Figure** **1**a, a series of acetates were selected as the reaction precursors, owing to their relatively high solubility, and the corresponding double perovskite structure were demonstrated, wherein [NaCl_6_]^5−^ and [InCl_6_]^3−^/[SbCl_6_]^3−^ octahedra are alternately arranged, and Cs^+^ ions occupy the center of the octahedral cavities. Specific details of synthesis are given in the Supporting information. Notably, the optimized Cs_2_NaInCl_6_:Sb^3+^ NCs were selected as hosts for the incorporation of RE ions, wherein the feeding ratio between Sb(OAc)_3_ and In(OAc)_3_ was tailored (Figure S1, Supporting Information). Subsequently, Cs_2_NaInCl_6_:Sb^3+^ NCs and RE ions (Sm^3+^, Eu^3+^, Tb^3+^, Dy^3+^)‐doped Cs_2_NaInCl_6_:Sb^3+^ NCs were characterized using transmission electron microscopy (TEM) for size and morphology, as shown in Figure 1b–f. These NCs have a similar cubic shape, high crystallinity, and good uniformity. Additionally, the particle size distributions are displayed in Figure S2 (Supporting Information), wherein the average sizes are around 10 nm. X‐ray diffraction (XRD) patterns of the as‐prepared samples are demonstrated in Figure 1g, suggesting that the Cs_2_NaInCl_6_ perovskite structure was formed and RE ions (Sm^3+^, Eu^3+^, Tb^3+^, and Dy^3+^) were incorporated into Cs_2_NaInCl_6_:Sb^3+^ NCs. All the NCs possessed the same cubic phase with no extra peaks after incorporating RE ions (Sm^3+^, Eu^3+^, Tb^3+^, and Dy^3+^), indicating the introduction of RE ions did not change the crystalline phase but only shift the diffraction peak of (220) toward smaller angle (on the right side of Figure 1g). Besides, based on the Bragg's law, we also calculated the lattice constants, and the calculated results were listed in Table S2 (Supporting Information). Considering the difference among the radii of Sm^3+^(0.964 Å), Eu^3+^(0.950 Å), Tb^3+^(0.923 Å), Dy^3+^(0.908 Å), and In^3+^ (0.810 Å), the expanded lattice preliminarily suggested that RE ions were introduced into the halide double perovskite lattice and occupied the In^3+^ site.^[^ ^12^ ^]^

High‐resolution TEM (HR‐TEM) images are demonstrated in Figure 1h,i, and Figure S3 (Supporting Information). The lattice constants of pure Cs_2_NaInCl_6_ NCs, Cs_2_NaInCl_6_:Sb^3+^ NCs and Tb^3+^‐doped Cs_2_NaInCl_6_:Sb^3+^ NCs were determined to be 3.69, 3.70, and 3.75 Å, respectively, corresponding to the (220) plane, indicating Tb^3+^ entered the double perovskite lattice. Moreover, as shown in Figure 1j, the selected area electron diffraction (SAED) pattern of Tb^3+^‐doped Cs_2_NaInCl_6_:Sb^3+^ NCs display the (220) and (400) cubic plane, further suggesting the formation of the double perovskite structure. Energy‐dispersive X‐ray spectroscopy (EDS) and element mapping images show that the lanthanide element presented in halide double perovskite NCs (Figures S4 and S5, Supporting Information). Furthermore, XPS analysis was carried out to confirm the presence of Tb element in Tb^3+^‐doped Cs_2_NaInCl_6_:Sb^3+^ NCs. The survey XPS spectra demonstrate that both the pristine Cs_2_NaInCl_6_:Sb^3+^ NCs and Tb^3+^‐doped Cs_2_NaInCl_6_:Sb^3+^ NCs comprise of Cs, Na, In, Sb, and Cl elements (Figure S6, Supporting Information). The high‐resolution XPS spectra show the presence of Tb element, and uncover that the binding energies of In 3d and Cl 2p varied after introducing Tb^3+^ ions, which robustly suggest that Tb^3+^ entered the lattice of halide double perovskite NCs, and substituted In^3+^ sites. The atom ratios obtained by XPS and EDS measurements in the doped samples are displayed in Table S3 (Supporting Information), which are close to the ICP‐MS measurement results (Table S4, Supporting Information); these experimental results together substantiate the successful doping of RE cations.

**Figure 2:**
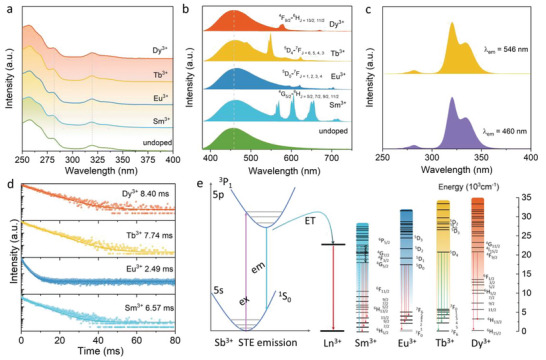
a) Absorption spectra and b) emission spectra of pristine Cs_2_NaInCl_6_:Sb^3+^ NCs and RE ions (Sm^3+^, Eu^3+^, Tb^3+^, Dy^3+^)‐doped Cs_2_NaInCl_6_:Sb^3+^ NCs. c) PLE spectra of Tb^3+^‐ion‐doped Cs_2_NaInCl_6_:Sb^3+^ NCs. d) Emission decay curves of RE ions (Sm^3+^, Eu^3+^, Tb^3+^, and Dy^3+^) emission monitored at corresponding main emission wavelengths. e) Sb^3+^ STE emission, the energy level of RE ions (Sm^3+^, Eu^3+^, Tb^3+^, and Dy^3+^) and the proposed photoluminescence mechanisms.

The optical properties of pristine Cs_2_NaInCl_6_:Sb^3+^ NCs and RE ions (Sm^3+^, Eu^3+^, Tb^3+^, and Dy^3+^)‐doped Cs_2_NaInCl_6_:Sb^3+^ NCs are displayed in **Figure** **2**. The absorption spectra of these samples are identical (Figure 2a), wherein the absorption peaks at 320 and 280 nm correspond to the transition of Sb^3+ 1^S_0_–^3^P_1_ and ^1^S_0_–^3^P_2_, respectively. ^[^ ^10^, ^13^ ^]^ The PL features are revealed via photoluminescence spectroscopy (Figure 2b). Under the excitation of 320 nm irradiation, the emission spectra of these samples showed a broad blue emission at around 460 nm corresponding to the Sb^3+^ STE emission, and the PL peak position appeared red‐shifted upon the introduction of different RE ions compared with the PL peak of pristine Cs_2_NaInCl_6_:Sb^3+^ NCs (455 nm), which may be related to the structural distortions caused by doping. Simultaneously, several components related to the intrinsic 4f–4f transition of RE ions were observed: ^4^G_5/2_–^6^H*_J_* (*J* = 5/2, 7/2, 9/2, 11/2) for Sm^3+^, ^5^D_0_–^7^F*_J_* (*J* = 1, 2, 3, 4) for Eu^3+^, ^5^D_4_–^7^F*_J_* (*J* = 6, 5, 4, 3) for Tb^3+^, and ^4^F_9/2_–^6^H*_J_* (*J* = 13/2, 11/2) for Dy^3+^. To uncover the origin of RE ions emission, taking Tb^3+^ incorporation as an example, the excitation spectra by selectively monitored at 460 nm (Sb^3+^ STE emission) and 546 nm related to the ^5^D_4_–^7^F_5_ transition of Tb^3+^ ions in Tb^3+^‐doped Cs_2_NaInCl_6_:Sb^3+^ NCs were measured, as shown in Figure 2c. These excitation spectra exhibit the same excitation bands, suggesting the energy transfer occurs from the Sb^3+^ STE emission to RE ions. The PLQYs of these samples are displayed in Table S5 (Supporting Information). Intriguingly, after incorporating RE ions, although the energy transfer process between Sb^3+^ STE emission and RE ions occurs, the Sb^3+^ STE emission intensity slightly increases, which indicates that there are two competitive pathways that influence Sb^3+^ STE emission after introducing RE ions. One is the energy transfer from the STE emission to the RE ions, which is considered to decrease the STE emission of host. The other is the passivation effect. It is reported that In^3+^ vacancy defect is found to be a deep defect in halide double perovskites, and isovalent doping helps to reduce vacancy defects in perovskite.^[^ ^4^ ^]^ Hence, the doping of isovalent RE ions can passivate defect and suppress the nonradiative recombination, enhancing the STE emission of host.

The PL decay curves of pristine Cs_2_NaInCl_6_:Sb^3+^ NCs and RE ions‐doped Cs_2_NaInCl_6_:Sb^3+^ NCs were determined by time‐resolved spectra, as exhibited in Figure S7 (Supporting Information), and the lifetime values are listed in Table S5 (Supporting Information). It is obvious that the average lifetimes of Sb^3+^ STE emission change slightly in RE ions‐doped Cs_2_NaInCl_6_:Sb^3+^ NCs, revealing the possible presence of two competitive pathways. Besides, the PL decay curves of RE ions in halide double perovskite NCs were measured, as shown in Figure 2d. The decay time for RE ions displays a single exponential behavior, which is coincident with the previous reports for the intrinsic transitions of RE ions, suggesting that RE ions are incorporated into the lattice of halide double perovskite NCs.^[^ ^14^ ^]^ Based on the above investigations, the corresponding energy transfer mechanism between the Sb^3+^ STE emission and RE ions is presented in Figure 2e. Under the excitation of 320 nm, the electrons in the ground state ^1^S_0_ were excited to the excited state ^3^P_1_, via the lattice relaxation, generating the broadband STE emission ascribed to the transition of ^3^P_1_‐^1^S_0_, and part of the energy transmitted to the emission level of RE ions via the nonradiative relaxation, further generating the corresponding RE ions emissions.

**Figure 3:**
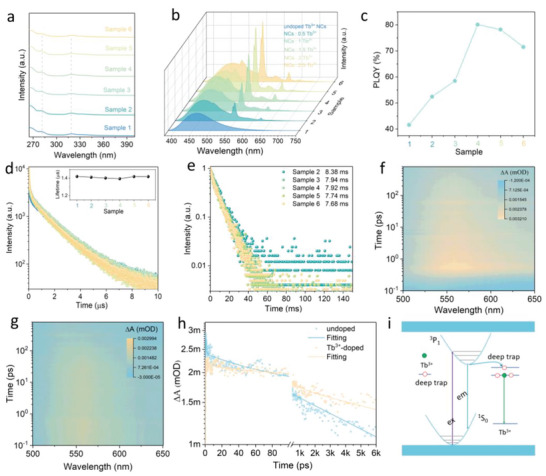
a) Absorption and b) PL spectra of Cs2NaInCl6:Sb^3+^ NCs and Tb^3+^‐doped Cs_2_NaInCl_6_:Sb^3+^ NCs with different amounts of Tb^3+^. c) PLQY values of six samples. d) Lifetime decay curves for six samples monitored at the Sb^3+^ STE emission. e) The lifetime decay curves of six samples monitored at the Tb^3+^ 546 nm emission. f,g) Pseudocolor Fs‐TA plot of Cs_2_NaInCl_6_:Sb^3+^ NCs and Tb^3+^‐doped Cs_2_NaInCl_6_:Sb^3+^ NCs. h) Normalized PIA decay curves of Cs_2_NaInCl_6_:Sb^3+^ NCs and Tb^3+^‐doped Cs_2_NaInCl_6_:Sb^3+^ NCs. i) Schematic PL mechanism of Tb^3+^‐doped Cs_2_NaInCl_6_:Sb^3+^ NCs, and the double functions of Tb^3+^ were emphasized.

The amount of RE ions has a crucial impact on the emission intensity of RE ions‐doped samples.^[^ ^15^ ^]^ We further investigated the doping amount effect on Tb^3+^‐doped Cs_2_NaInCl_6_:Sb^3+^ NCs. Six samples (denoted as samples 1–6) with varied Tb^3+^ ion doping amount by changing the feeding amount of Tb(OAc)_3_·H_2_O (0, 0.25, 0.5, 0.75, 1, and 1.25 mmol). The crystalline structure is consistent for the NCs doped with different concentrations (ICP‐MS in Table S4 in the Supporting Information for actual doping concentrations), while the lattice constants of double perovskite nanocrystals increased with the doping concentration that were verified via the XRD and HR‐TEM measurements (Figure S8 and Table S2, Supporting Information). Meanwhile, there is no apparent change in the morphology and size of the NCs doped with different concentrations. Furthermore, the optical properties of the six samples were studied, as shown in **Figure** **3**. UV–vis absorption measurements displayed that all six samples show similar absorption spectral profiles (Figure 3a). The emission spectra for Tb^3+^ ion‐doped halide double perovskite NCs with different amounts demonstrate the presence of Sb^3+^ STE emission of host and intrinsic Tb^3+^ ion ^5^D_4_–^7^F*_J_* (*J* = 6, 5, 4, 3), as shown in Figure 3b. The PLQYs of the six samples were measured (Figure 3c). As a function of Tb^3+^ ion concentration, the overall PLQY initially increased, approached the highest, and then decreased with more Tb^3+^ doping amount. The optimized overall PLQY was 80.1% in 1.5Tb^3+^‐doped Cs_2_NaInCl_6_:Sb^3+^ NCs. Moreover, the STE emission intensity of Tb^3+^‐doped Cs_2_NaInCl_6_:Sb^3+^ NCs apparently increased, compared with the host Cs_2_NaInCl_6_:Sb^3+^ NCs, suggesting the presence of a passivation effect (Figure S9, Supporting Information). Time‐resolved spectra measurements for STE emission were carried out for the six samples, and the fitting lifetime results are demonstrated in Figure 3d. Upon increasing the Tb^3+^ doping amount, the STE lifetime value showed no apparent changes (Figure 3d). The PL lifetimes of Tb^3+^ were demonstrated in Figure 3e, and the lifetime values decreased from 8.38 to 7.68 ms with the increase of the doping amount of Tb^3+^, which is consistent with a previous report.^[^ ^9b^ ^]^

Fs‐TA measurement could supply in‐depth information for the nonradiative carrier‐trapping and energy transfer processes.^[^ ^16^ ^]^ To further investigate the RE ions effect on the host of Cs_2_NaInCl_6_:Sb^3+^ NCs, Fs‐TA spectroscopy measurement was performed for the samples of pristine Cs_2_NaInCl_6_:Sb^3+^ NCs, and the optimized Tb^3+^‐doped Cs_2_NaInCl_6_:Sb^3+^ NCs. Both Cs_2_NaInCl_6_:Sb^3+^ NCs and Tb^3+^‐doped Cs_2_NaInCl_6_:Sb^3+^ NCs display broad photoinduced absorption (PIA) signals ranging 500 to 650 nm (Figure 3f,g), which provide direct evidence for the formation of Sb^3+^ STE emission. Additionally, we compared the PIA decay dynamics for Cs_2_NaInCl_6_:Sb^3+^ NCs and Tb^3+^‐doped Cs_2_NaInCl_6_:Sb^3+^ NCs (Figure 3h). The decay of the PIA signal in the Cs_2_NaInCl_6_:Sb^3+^ NCs exhibited three different components with different time scales. The ultrafast decays with *τ*_1_ = 1.5 ps (12.3%), and *τ*_2_ = 50.0 ps (23.7%) are ascribed to the volume defect trapping and surface defect trapping, respectively. ^[^ ^2b^ ^]^ The slow component with *τ*_3_ longer than 6 ns (64.0%) can be assigned to the intrinsic lifetime of excited STE emission. For the Tb^3+^‐doped Cs_2_NaInCl_6_:Sb^3+^ NCs, four different time components could be found. The three ultrafast lifetimes *τ*_1_ = 1.5 ps (6.9%), *τ*_2_ = 47.2 ps (9.3%), and *τ*_3_ > 6 ns (69.8%) possess the similar origin to the Cs_2_NaInCl_6_:Sb^3+^ NCs, owing to the semblable time constants. Moreover, an additional component with the time constant of *τ*_4_ = 500 ps in the Tb^3+^‐doped Cs_2_NaInCl_6_:Sb^3+^ NCs exhibits the energy transfer between the Sb^3+^ STE emission and Tb^3+^ ions. ^[^ ^16^, ^17^ ^]^ Notably, the amplitudes of *τ*_1_ and *τ*_2_ decreased, compared with the Cs_2_NaInCl_6_:Sb^3+^ NCs, which corresponds to the enhancement of STE emission after introducing Tb^3+^ ions. Based on the above discussion, a profound PL mechanism in Tb^3+^‐doped Cs_2_NaInCl_6_:Sb^3+^ NCs is proposed (Figure 3i), wherein the double functions of Tb^3+^ ions are emphasized. With the excitation of 320 nm, the STE emission generated with 460 nm broad emission. Meanwhile, the energy transfer occurs between STE emission and Tb^3+^ within 500 ps, wherein the Tb^3+^ can fill in the vacancy defect and subsequently is excited, generating the corresponding Tb^3+^ ions emission.

Additionally, the concentration‐dependent XRD patterns and PL spectra for the Dy‐doped and Eu‐doped double perovskite nanocrystals were systematically studied, as shown in Figure S10 (Supporting Information). The PLQYs of concentration‐optimized double perovskite nanocrystals have exceeded 60%.

The stability of the sample is closely related to the applications and hence was further investigated. Taking Tb^3+^‐doped Cs_2_NaInCl_6_:Sb^3+^ NCs for an example, the sample's XRD pattern, PL spectrum, and PL intensity remained almost unchanged after stored in air for six months, suggesting that the sample possesses excellent stability (Figures S11 and S12, Supporting Information).

**Figure 4:**
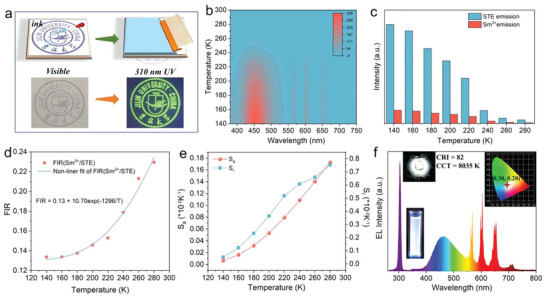
a) Schematic diagram of the screen printing for anti‐counterfeiting application with Tb^3+^‐doped Cs_2_NaInCl_6_:Sb^3+^ NCs. b) Contour map of thermal evolution PL spectrum of Sm^3+^‐doped Cs_2_NaInCl_6_:Sb^3+^ NCs under 320 nm excitation in the range of 140–280 K. c) Corresponding histograms of integrated intensity of Sb^3+^ STE emission and Sm^3+^ emission at various temperatures. d) Fitting curve of temperature‐dependent FIR (*I*_Sm_/*I*_STE_). e) Absolute sensitivity (Sa) and relative sensitivity (Sr) verse absolute temperature for Sm^3+^‐doped Cs_2_NaInCl_6_:Sb^3+^ NCs under 320 nm excitation. f) PL spectra of WLED based on Sm^3+^‐doped Cs_2_NaInCl_6_:Sb^3+^ NCs and 310 nm LED chip (the insets are the pictures of white‐emitting Sm^3+^‐doped Cs_2_NaInCl_6_:Sb^3+^ NCs under UV excitation, WLED in operation, and CIE chromaticity coordinates diagram of WLED).

Benefitting from the excellent optical properties and great stability of RE ions‐doped Cs_2_NaInCl_6_:Sb^3+^ NCs, a few luminescence‐related applications were demonstrated. **Figure** **4**a illustrates a screen‐printing utilizing Tb^3+^‐doped Cs_2_NaInCl_6_:Sb^3+^ NCs as the ink. Under natural light, no colorful logo pattern was observed. Significantly, the green‐emitting logo was seen under the 310 nm light source, which can be applied in encryption applications.

Temperature sensitivity of the STE emission and RE ions emission is different, inspiring us to utilize the RE‐ion‐doped Cs_2_NaInCl_6_:Sb^3+^ NCs for temperature sensing. Here, Sm^3+^‐doped Cs_2_NaInCl_6_:Sb^3+^ NCs were selected for optical thermometry. Figure 4b displays the contour map of thermal evolution PL spectra of Sm^3+^‐doped Cs_2_NaInCl_6_:Sb^3+^ NCs in the range of 140–280 K. The emission peak positions displayed no change, but the emission intensity decreased, which was originated from the thermal quenching effect. Besides, the integral intensity of Sb^3+^ STE and Sm^3+^ emissions at different temperatures is demonstrated in Figure 4c. It is evident that the Sb^3+^ STE emission drops much faster than the Sm^3+^ emission; namely, the STE emission is more sensitive to the temperature, which is consistent with the insensitivity of RE ions to temperature.^[^ ^18^ ^]^ The fluorescence intensity ratio (FIR) technology was utilized to determine whether the prepared sample was successfully applied to actual temperature measurement. The relationship between temperature and FIR can be described via the equations^[^ ^19^ ^]^

(1) $$\begin{array}{ccc} \mathrm{FIR}\; & = & \frac{I_{\mathrm{Sm}}}{I_{\mathrm{STE}}}\;=\frac{I_{0,\;\mathrm{Sm}}}{I_{0,\;\mathrm{STE}}}\frac{1+A_{\mathrm{STE}\;}\exp\left(-\Delta E_{\mathrm{STE}}/k_{B}T\right)}{1+A_{\mathrm{Sm}}\exp\left(-\Delta E_{\mathrm{Sm}}/k_{B}T\right)}\\ & \approx & B+C\;\exp\left(-\Delta E/k_{B}T\right) \end{array}$$

where the constants *B*, *C*, and Δ*E* are related to Sb^3+^ and Sm^3+^ ions. Figure 4d displays the relationship between FIR (*I*_Sm3+_/*I*_STE_) and the absolute temperature *T*. It was found that the value of FIR increased from 0.133 to 0.220, along with the temperature changed from 140 K to 280 K. In the temperature sensing field, the absolute temperature sensitivity (Sa) and the relative temperature sensitivity (Sr) are crucial parameters to evaluate the quality of optical thermometry. The values of Sa and Sr can be calculated via the following expressions^[^ ^20^ ^]^

(2) $$\mathrm{Sa}\;=\left|\frac{\partial \mathrm{FIR}}{\partial T}\right|=C\;\exp\left(-\Delta E/k_{B}T\right)\;\times \frac{\Delta E}{k_{B}T^{2}}$$

(3) $$\begin{array}{ccc} \mathrm{Sr}\; & = & \;100\%\;\times \left|\frac{1}{\mathrm{FIR}}\frac{\partial \mathrm{FIR}}{\partial T}\right|\\ & = & \;100\%\;\times \frac{C\;\exp\left(\frac{-\Delta E}{k_{B}T}\right)}{B+C\;\exp\left(\frac{-\Delta E}{k_{B}T}\right)}\times \frac{\Delta E}{k_{B}T^{2}} \end{array}$$

The calculated Sa and Sr via the above formulas are demonstrated in Figure 4e. The maximum values of Sa and Sr of the samples reached as high as 0.173 and 0.753% K^−1^, respectively. Furthermore, temperature resolution (*δT*) is another important parameter to evaluate the performance of optical thermometry, which can be expressed as follows^[^ ^21^ ^]^

(4) $$\delta T\;=\frac{1}{\mathrm{Sr}}\frac{\Delta \mathrm{FIR}}{\mathrm{FIR}}$$

In this case, the ΔFIR/FIR stands for the relative error in the measurement process, and its value is approximately 0.5%.^[^ ^22^ ^]^ Hence, it is calculated that the minimum temperature resolution of 0.64 at 280 K. Compared previously reported temperature sensors based on other emissive materials, RE ions doped halide double perovksite NCs exhibited great temperature sensing performance (Table S6, Supporting Information). The above results demonstrated that the Sm^3+^‐doped Cs_2_NaInCl_6_:Sb^3+^ NCs are potential materials for a noncontact optical thermometer.

In addition to the optical thermometry applications, Sm^3+^ ion‐doped halide double perovskite NCs also showed a great potential in the field of WLEDs. Single‐component white‐emitting materials are of particular interest owing to the simplicity of device structure and the avoidance of the self‐absorption and color instability.^[^ ^23^ ^]^ Notably, Sm^3+^‐doped Cs_2_NaInCl_6_:Sb^3+^ NCs demonstrated an efficient and stable white light emission under the excitation of 320 nm. The WLED was fabricated by combining the 310 nm LED chip and white‐emitting Sm^3+^‐doped Cs_2_NaInCl_6_:Sb^3+^ NCs@PMMA composite materials. The luminescence spectrum and luminescence photograph of the WLED are shown in Figure 4f. At a voltage of 5.5 V, the WLEDs emit white light with CIE color coordinates of (0.30, 0.28), a luminous efficiency of 37.5 lm W^−1^, a CCT of 8035 K, and a CRI of 82. Furthermore, Table S7 (Supporting Information) lists some typical WLED performance fabricated by 310 nm UV chip, displaying that Sm^3+^‐doped Cs_2_(Na/Sb)InCl_6_ NCs possess a great prospect in WLED application.

## 3 Conclusions

In summary, RE ions (Sm^3+^, Eu^3+^, Tb^3+^, and Dy^3+^)‐doped Cs_2_NaInCl_6_:Sb^3+^ NCs were synthesized via hot injection method and displayed the broad Sb^3+^ STE emission and RE ions emission. Further, the doping amount of Tb^3+^ effect on the optical properties in Tb^3+^‐doped Cs_2_NaInCl_6_:Sb^3+^ NCs was systematically investigated, and the optimized samples exhibited a PLQY of 80.1%. The Fs‐TA measurements were performed for Cs_2_NaInCl_6_:Sb^3+^ NCs and Tb^3+^‐doped Cs_2_NaInCl_6_:Sb^3+^ NCs, and the double functions of Tb^3+^ were investigated. Based on the excellent optical characteristic and great stability of RE ions‐doped halide double perovskite NCs, the anti‐counterfeiting, optical thermometry, and WLED applications were demonstrated with outstanding performances. This work provides the environment‐friendly and efficiently luminescent candidates for the perovskite multifunctional applications.

## Conflict of Interest

The authors declare no competing financial interest.

## Supporting information

Supporting Information

## Data Availability

The data that support the findings of this study are available from the corresponding author upon reasonable request.
