# Supplementary material for: Halide Double Perovskite Nanocrystals Doped with Rare‐Earth Ions for Multifunctional Applications
Source: Adv Sci (Weinh). 2023 Apr 28;10(20):2207571. doi: 10.1002/advs.202207571 (PMC10369281; doi:10.1002/advs.202207571)
Supplement: Supplementary file 1 — Supporting Information [file ADVS-10-2207571-s001.pdf]

ADVANCED SCIENCE
Open Access

Supporting Information

for *Adv. Sci.*, DOI 10.1002/advs.202207571

Halide Double Perovskite Nanocrystals Doped with Rare-Earth Ions for Multifunctional Applications

Xin Li, Dingdi Wang, Yuan Zhong, Feng Jiang, Deqiang Zhao, Siqi Sun, Po Lu, Min Lu, Zhenyu Wang, Zhennan Wu, Yanbo Gao, Yu Zhang*, William W. Yu* and Xue Bai**

Supporting Information

Halide double perovskite nanocrystals doped with rare-earth ions for multifunctional applications

Xin Li, Dingdi Wang, Yuan Zhong, Feng Jiang, Deqiang Zhao, Siqi Sun, Po Lu, Min Lu, Zhenyu Wang, Zhennan Wu, Yanbo Gao, Yu Zhang*, William W. Yu*, and Xue Bai**

1. Experimental Section

1.1 Materials:

Cesium acetate (CsOAc, 99.99%, Aladdin), sodium acetate (NaOAc, 99.99%, anhydrous, Aladdin), indium acetate ($In(OAc)_3$, 99.99%, Alfa Aesar), antimony acetate ($Sb(OAc)_3$, 99.9%, Alfa Aesar), europium acetate hydrate ($Eu(OAc)_3·H_2O$, 99.99%, Aladdin), samarium acetate hydrate ($Sm(OAc)_3·H_2O$, 99.9%, Aladdin), terbium acetate hydrate ($Tb(OAc)_3·H_2O$, 99.9%, Aladdin), dysprosium(III) acetate tetrahydrate ($Dy(OAc)_3·4H_2O$, 99.9%, Aladdin), chlorotrimethylsilane (TMS-Cl, 98%, Alfa Aesar), 1-octadecene (90%, Aladdin), oleylamine (OLA, 80%, Alfa Aesar), oleic acid (OA, 90%, Sigma-Aldrich), toluene (99.5%, Sinopharm Chemical Reagent Co., Ltd, China), and n-hexane (97%, Aladdin) were used directly without further purification.

1.2 Synthesis of $Cs_2NaInCl_6$ NCs and $Cs_2NaInCl_6$: Sb^{3+} NCs

The $Cs_2NaInCl_6$ NCs was synthesized by a reported hot injection method.[1] CsOAc (0.1247 g), NaOAc (0.369 g), $In(OAc)_3$ (0.144 g), OA (2.8 ml), OLA (0.7 ml), and 1-octadecene (10 ml) were added into a three-necked flask and stirred under vacuum condition at 110 °C for 1 h. Then, the mixture solution was set at 180 °C under a nitrogen atmosphere, and TMS-Cl

(0.4 mL) was swiftly injected at 165 °C. When the reaction mixture's temperature increased to 175 °C, the above solution was immediately cooled with an ice-water bath. The $Cs_2NaInCl_6$ NCs were obtained via three centrifugation process. The NCs in the mixture solution were centrifuged at 9000 rpm for 20 min, then the supernatant was discarded. The precipitate was dispersed in toluene, and centrifuged at 10000 rpm for 15 min, and the supernatant was discarded again, and the as-prepared samples were freeze-dried. Then, the obtained precipitate was dissolved to n-hexane, and centrifuged at 6000 rpm for 15 min. The supernatant containing $Cs_2NaInCl_6$ NCs were obtained. As for $Cs_2NaInCl_6$: Sb^{3+} NCs, the synthesis process was identical to the synthesis of $Cs_2NaInCl_6$ NCs, but replaced 0.5 mmol $In(OAc)_3$ (0.144 g) with 0.25 mmol $Sb(OAc)_3$ (0.0747 g) and 0.25 mmol $In(OAc)_3$ (0.072 g). The optimal ratio of $Sb(OAc)_3$ and $In(OAc)_3$ was ascertained via the concentrations-dependent on PL spectra, wherein the ratio of $Sb(OAc)_3$ and $In(OAc)_3$ as 1:1, the PLQY was the highest (**Figure S1**).

1.3 Synthesis of RE ions doped $Cs_2NaInCl_6$: Sb^{3+} NCs

The RE ions doped $Cs_2NaInCl_6$: Sb^{3+} NCs were synthesized using the same method employed for the undoped and Sb^{3+}-doped $Cs_2NaInCl_6$ NCs with minor modifications. Additional lanthanide acetate (0.95 mmol) was casted to the raw materials, and all the other reaction parameters were the same. For different amounts Tb^{3+}-doped $Cs_2NaInCl_6$: Sb^{3+} NCs, 0.25, 0.5, 0.75, 1, and 1.25 mmol $Tb(OAc)_3·H_2O$ were casted to the raw materials, respectively, and all the other reaction parameters were the same.

1.4 LED fabrication

The powder of Sm^{3+}-doped $Cs_2NaInCl_6$: Sb^{3+} NCs were dispersed in a transparent PMMA/toluene solution and stirred for 2 h and then coated onto a 310 nm UV LED chip. The LEDs from Sm^{3+}-doped $Cs_2NaInCl_6$: Sb^{3+} NCs were thus fabricated after drying of the color conversion layer.

1.5 Characterizations

For TEM measurement, JEM-2100F at an accelerating voltage of 200 kV was used. XRD measurements were performed using a Bruker D8 Advance X diffractometer (Cu Kα, λ = 1.5406 Å). High-resolution TEM (HRTEM) images and EDS elemental mapping images were collected with a FEI Tecnai F20. X-ray photoelectron spectroscopy (XPS) measurements were carried on a Thermo ESCALAB 250 spectrometer equipped with a monochromatic Al Kr radiation source (1486.6 eV).

Absorption spectra were collected using a Shimadzu UV-2550 spectrophotometer. Photoluminescence (PL) and photoluminescence excitation (PLE) spectra were measured on a QM8000 HORIBA spectrometer. The PLQY measurements were carried out using QM8000 HORIBA spectrometer with an integrating sphere. The PL lifetimes of Sb^{3+} STE emission were measured using a time-correlated single-photon counting (TCSPC) lifetime spectroscopy system with a nano-LED (EPL-340 nm) as the single wavelength excitation light source. The PL lifetimes of Ln ions were measured using a laser-system consisting of a Nd:YAG pumping laser (1064 nm), a third-order Harmonic-Generator (355 nm) and a tunable optical parameter oscillator (OPO, Continuum Precision II 8000) with a pulse duration of 10 ns, a repetition frequency of 10 Hz and a line width of 4-7 cm^{-1}. The ultrafast Fs-TA measurements were conducted under a pump wavelength of 340 nm on HELIOS (Ultrafast systems) spectrometer. The PL spectra of the WLED was measured using a programmable source meter (Keithley model 2400) and a luminance meter/spectrometer (PhotoResearch PR665).

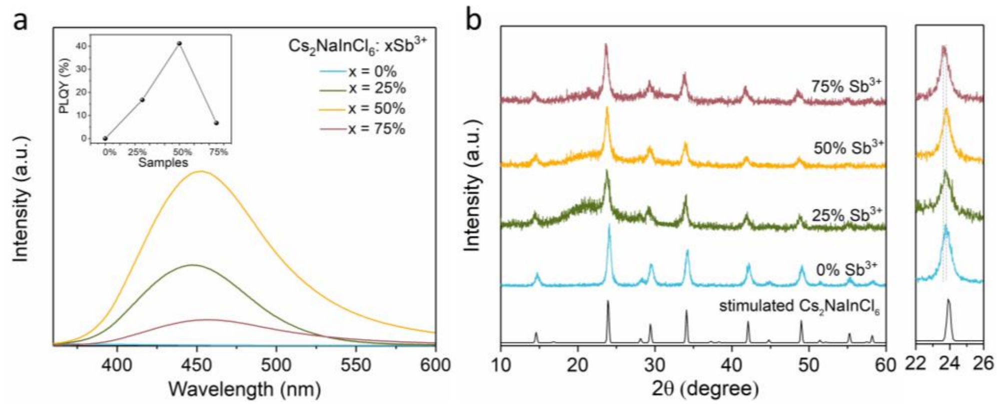

Figure S1. a) PL spectra of $Cs_2NaInCl_6$ NCs doped with different amounts of Sb^{3+}. Inset is the corresponding PLQYs. b) XRD patterns of $Cs_2NaInCl_6$ doped with different amount of Sb^{3+}.

The optimal doping concentration of Sb^{3+} in Sb^{3+}-doped $Cs_2NaInCl_6$ NCs was determined via the concentration-dependent PL spectra. As displayed in Figure S1a, the PL intensity first increased and then decreased, and the PL emission intensity was the highest when the doping ratio was 50%. Moreover, the actual doping amount was ascertained via ICP-MS (Table S1), wherein the actual doping amount was lower than the feeding ratio. Figure S1b demonstrates the XRD patterns of $Cs_2NaInCl_6$ doped with different amounts of Sb^{3+}, which indicates the formation of halide double perovskite. Besides, it is found that the diffraction peak of the (220) plane slightly shifts toward smaller angle, suggesting that Sb^{3+}ions replaced In^{3+} ions because the ionic radius of Sb^{3+} (0.92 Å) is larger than that of In^{3+} (0.81 Å).

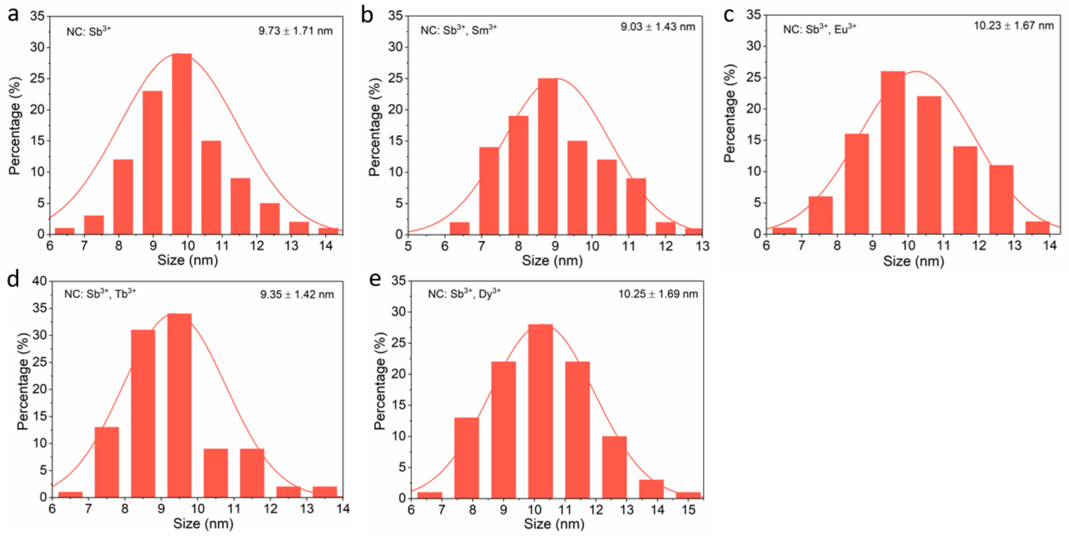

Figure S2. a-f) Particle size distributions of $Cs_2NaInCl_6$: Sb^{3+} NCs, and RE ions (Sm^{3+}, Eu^{3+}, Tb^{3+}, Dy^{3+}) incorporated $Cs_2NaInCl_6$: Sb^{3+} NCs.

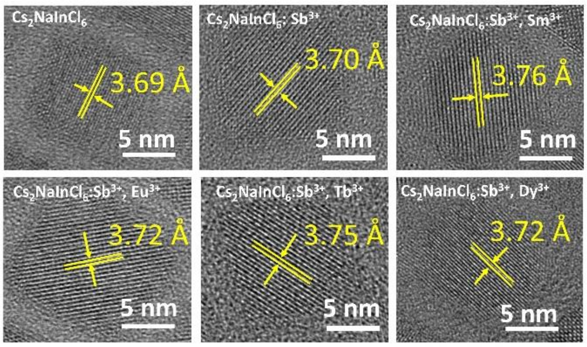

Figure S3. HR-TEM analysis of pure $Cs_2NaInCl_6$ and crystals with Sb and RE additions.

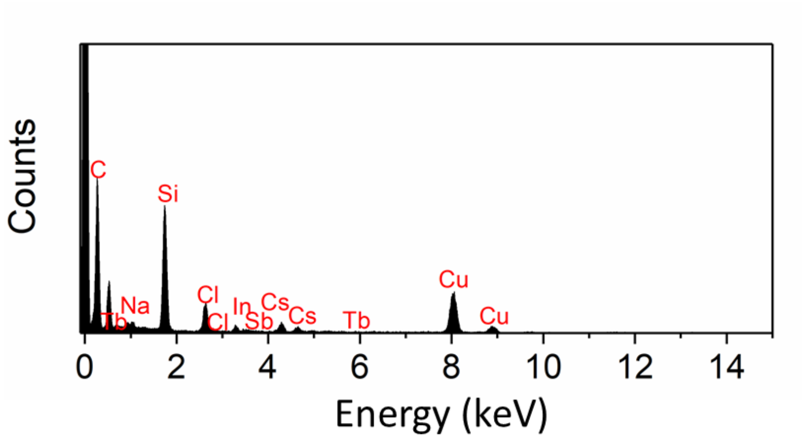

Figure S4. EDS of Tb^{3+} ion incorporated $Cs_2NaInCl_6$: Sb^{3+} NCs.

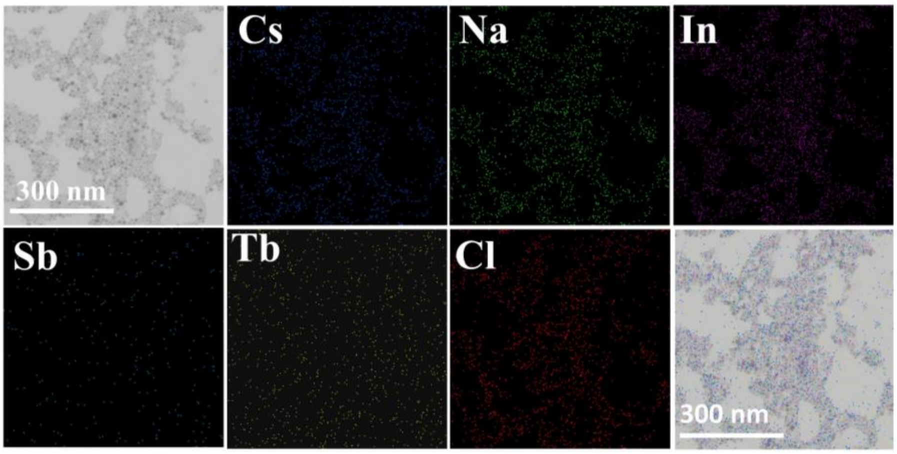

Figure S5. Elemental mappings of Cs, Na, In, Sb, Cl and Tb elements in the Tb^{3+} ion incorporated $Cs_2NaInCl_6$: Sb^{3+} NCs; the last frame on the row shows the overlapped TEM/elemental mapping image.

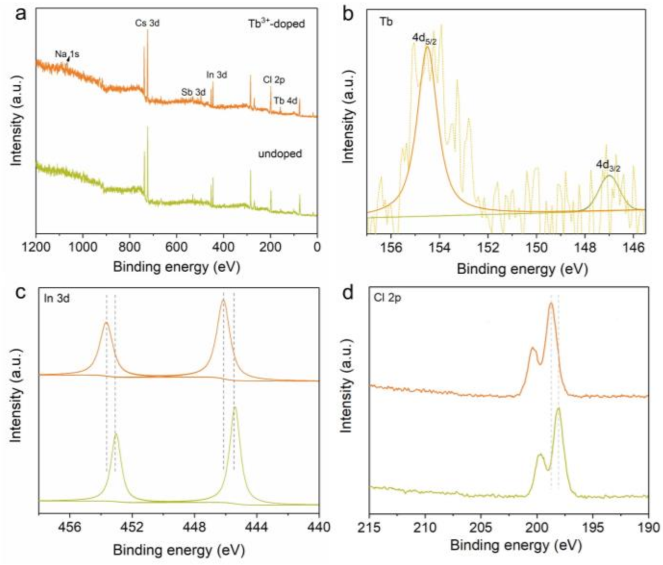

Figure S6. a) Survey XPS spectra for $Cs_2NaInCl_6$: Sb^{3+} NCs and Tb^{3+}-codoped $Cs_2NaInCl_6$: Sb^{3+} NCs. b-d) High-resolution XPS spectra of Tb 4d, In 3d and Cl 2p, respectively.

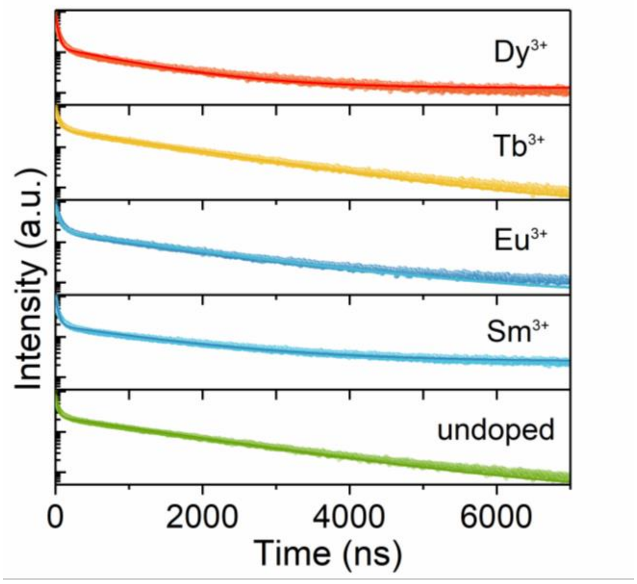

Figure S7. Emission decays of Sb^{3+} STE emission (monitored at 460 nm) for the $Cs_2NaInCl_6$: Sb^{3+} NCs and $Cs_2NaInCl_6$: Sb^{3+} NCs doped with different RE ions (Sm^{3+}, Eu^{3+}, Tb^{3+}, Dy^{3+}).

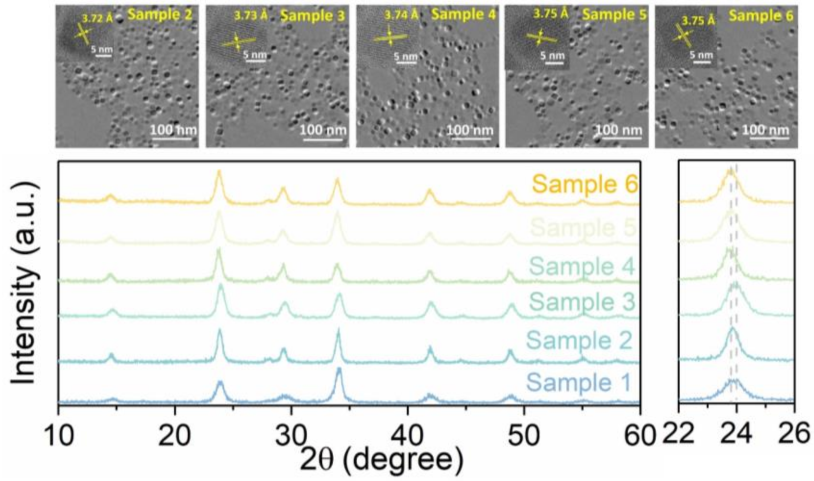

Figure S8. a) TEM and HR-TEM images; b) XRD patterns of the as-prepared Tb^{3+}-doped $Cs_2NaInCl_6$: Sb^{3+} with the different doping concentrations.

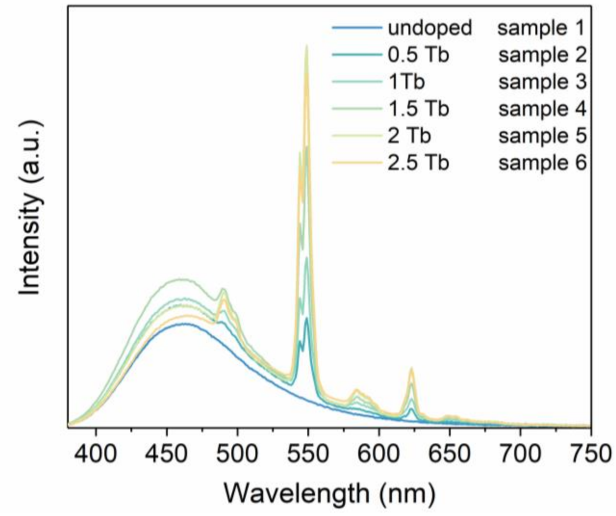

Figure S9. PL spectra of Tb^{3+}-doped $Cs_2NaInCl_6$: Sb^{3+} NCs with different amounts of Tb^{3+}.

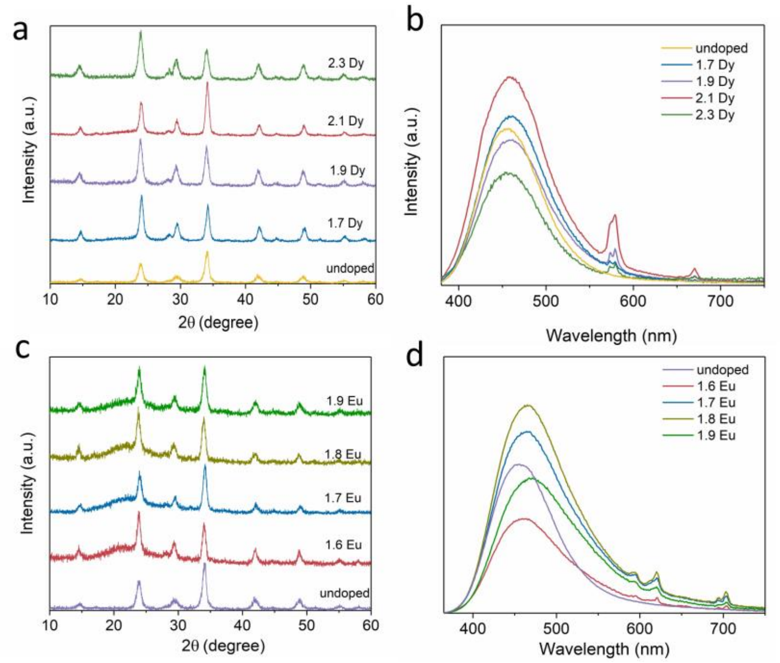

Figure S10. a-b) XRD patterns and PL spectra of Dy^{3+}-doped $Cs_2NaInCl_6$: Sb^{3+} NCs with different Dy^{3+} amounts; c-d) XRD patterns and PL spectra of Eu^{3+}-doped $Cs_2NaInCl_6$: Sb^{3+} NCs with different Eu^{3+} amounts.

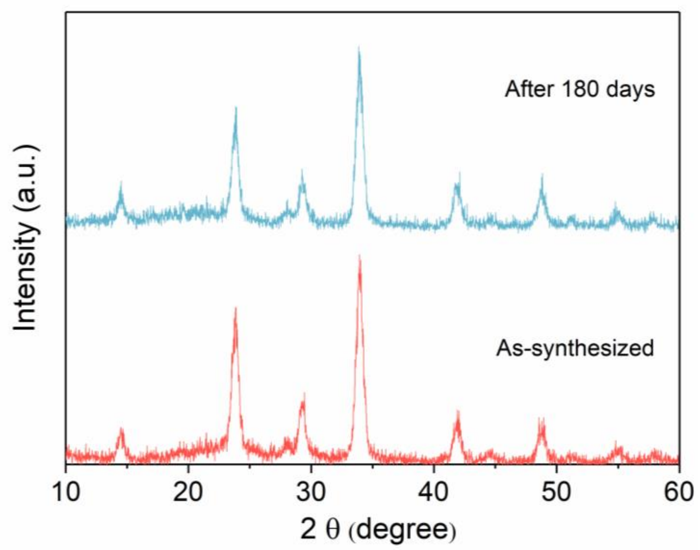

Figure S11. XRD patterns of Tb^{3+}-doped $Cs_2NaInCl_6$: Sb^{3+} NCs before and after their exposure to ambient air for 180 days.

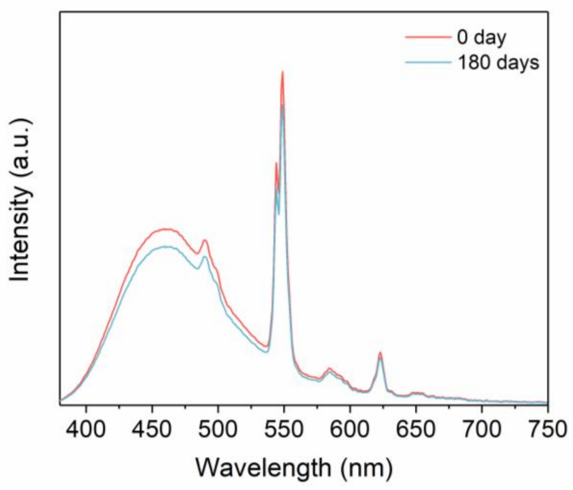

Figure S12. Comparison of PL spectra intensity of Tb^{3+}-doped $Cs_2NaInCl_6$: Sb^{3+} NCs before and after their exposure to ambient air for 180 days.

Table S1. ICP-MS results of Sb^{3+}-doped $Cs_2NaInCl_6$ NCs with different Sb^{3+} feeding ratios.

$Cs_2NaInCl_6$: xSb^{3+}	In (ppb)	Sb (ppb)	In (%)	Sb (%)
25%	377.64	8.72	97.85	2.15
50%	120.30	7.20	94.65	5.35
75%	105.32	22.43	83.18	16.82

Table S2. XRD scattering angles and lattice constants of pure $Cs_2NaInCl_6$ NCs, $Cs_2NaInCl_6$: Sb^{3+} NCs, RE^{3+} doped $Cs_2NaInCl_6$: Sb^{3+} NCs and Tb^{3+}-doped $Cs_2NaInCl_6$: Sb^{3+} NCs with different doping amounts.

Sample	2 Theta (degree)	Lattice constant (Å)
$Cs_2NaInCl_6$ NCs	24.04	3.69
$Cs_2NaInCl_6$:Sb^{3+} NCs	24.00	3.70
Sm^{3+}-doped $Cs_2NaInCl_6$:Sb^{3+} NCs	23.65	3.76
Eu^{3+}-doped $Cs_2NaInCl_6$:Sb^{3+} NCs	23.84	3.72
Tb^{3+}-doped $Cs_2NaInCl_6$:Sb^{3+} NCs	23.82	3.73
Dy^{3+}-doped $Cs_2NaInCl_6$:Sb^{3+} NCs	23.88	3.72
0.5 Tb^{3+} -doped $Cs_2NaInCl_6$:Sb^{3+}NCs	23.88	3.72
1.0 Tb^{3+} -doped $Cs_2NaInCl_6$:Sb^{3+}NCs	23.78	3.73
1.5 Tb^{3+} -doped $Cs_2NaInCl_6$:Sb^{3+}NCs	23.76	3.74

2.0Tb^{3+} -doped $Cs_2NaInCl_6$:Sb^{3+}NCs	23.73	3.75
2.5Tb^{3+} -doped $Cs_2NaInCl_6$:Sb^{3+}NCs	23.69	3.75

Table S3. Fitting results for the XPS patterns of element composition ratios and EDS analysis for RE doped $Cs_2NaInCl_6$: Sb^{3+} (RE = Sm^{3+}, Eu^{3+}, Tb^{3+}, Dy^{3+}) nanocrystals.

RE doped Sample	XPS (at%)						EDS (at%)					
	Cs	Na	In	Sb	RE	Cl	Cs	Na	In	Sb	RE	Cl
Sm^{3+}	18.5	8.6	11.0	0.8	1.6	59.5	18.7	8.3	10.5	0.8	1.5	60.2
Eu^{3+}	19.1	8.9	9.5	0.5	1.2	60.8	19.0	8.4	9.7	0.6	1.1	61.2
Tb^{3+}	20.0	8.8	10.3	0.6	1.9	58.4	20.3	8.6	10.0	0.7	1.8	58.6
Dy^{3+}	18.8	9.0	10.5	0.6	0.5	60.6	19.0	8.7	10.3	0.6	0.6	60.8

Table S4. Actual concentrations of rare earth dopants in the RE ions doped $Cs_2NaInCl_6$: Sb^{3+} NCs determined from ICP-MS data.

Sb/RE: $Cs_2NaInCl_6$	Nominal ratio					Actual content (ICP-MS)				
	Sb/ (In+Sb)	Sm/ (In+Sb)	Eu/ (In+Sb)	Dy/ (In+Sb)	Tb/ (In+Sb)	Sb^{3+} (%)	Sm^{3+} (%)	Eu^{3+} (%)	Dy^{3+} (%)	Tb^{3+} (%)
Sm (0.95 mmol)	0.5	1.9	/	/	/	5.05	1.47	/	/	/
Eu (0.95 mmol)	0.5	/	1.9	/	/	5.33	/	1.27	/	/
Dy (0.95 mmol)	0.5	/	/	1.9	/	5.21	/	/	0.58	/
Tb (0.95 mmol)	0.5	/	/	/	1.9	5.09	/	/	/	2.01
Tb (0.25 mmol)	0.5	/	/	/	0.5	4.99	/	/	/	0.52
Tb (0.5 mmol)	0.5	/	/	/	1	5.02	/	/	/	1.17
Tb (0.75 mmol)	0.5	/	/	/	1.5	5.00	/	/	/	1.64
Tb (1.0mmol)	0.5	/	/	/	2	4.89	/	/	/	2.10
Tb (1.25 mmol)	0.5	/	/	/	2.5	5.07	/	/	/	2.33

Table S5. Parameters for undoped and RE ions (Sm^{3+}, Eu^{3+}, Tb^{3+}, Dy^{3+}) doped $Cs_2NaInCl_6$: Sb^{3+} NCs with similar feeding ratio of 0.95 mmol.

	$Cs_2NaInCl_6$: Sb^{3+} NCs	NCs doped with different RE ions			
Doping ion	---	Sm^{3+}	Eu^{3+}	Tb^{3+}	Dy^{3+}
Radius of doping ion (Å)	---	0.964	0.950	0.923	0.908
PLQY (%)	41.2	65.0	45.0	59.2	43.1
STE lifetime (ns)	1419.00	1425.02	1394.22	1380.00	1465.51
Ln ions lifetime (ms)	---	6.57	2.49	7.74	8.40

Table S6. Summarized maximum absolute sensitivity (Max. Sa), maximum relative sensitivity (Max. Sr), and minimum temperature resolution (δT) values of previously reported Sm^{3+}-doped materials.

Material	Max.Sa (K^{-1})	Max.Sr (% K^{-1})	Temperature range (K)	Min δT (K)	Ref.
YVO_4 :Sm^{3+}	-	0.31	299-466	-	[2]
$GdVO_4$:Sm^{3+}	-	0.045	293-853	-	[3]
Ca_2LaNbO_6 :Sm^{3+}	-	0.23	313-573	2.19	[4]
YNbO4 :Sm^{3+}	0.0007	0.43	303-773	-	[5]
$Bi_2Ga_4O_9$:Cr^{3+}	-	0.70	80-450	-	[6]
$Sr_3Y_2Ge_3O_{12}$:Bi^{3+}, Sm^{3+}	0.0017	0.38	298-498	-	[7]
$Li_3Gd_3Te_2O_{12}$:Bi^{3+}, Pr^{3+}	-	0.672	100-300	-	[8]
$Ca_2YZr_2Al_3O_{12}$:Bi^{3+}, Eu^{3+}	0.00826	0.664	250-575	-	[9]
$SrAl_{12}O_{19}$:Mn^{4+}	0.004	0.27	273-393	-	[10]
$Cs_2WO_2F_4$:Mn^{4+}	-	0.21	10-433	-	[11]
$BaNb_2O_6$:Pr^{3+}	-	0.61	310-470	-	[12]
$LiY_9(SiO_4)_6O_2$: Ce^{3+}, Tb^{3+}	-	0.35	293–473	>0.02	[13]

$LiY_9(SiO_4)_6O_2$: Ce^{3+}, Dy^{3+}	-	0.43	300-400	-	[14]
$Cs_2NaInCl_6$: Sb^{3+}, Sm^{3+}	0.00173	0.75	140-280	0.64	This work

Table S7. Summarized WLED parameters fabricated by 310 nm UV chip.

WLED	CIE coordinate	CRI	CCT (K)	Luminous efficiency (lm/W)	Chip (nm)	Single-phase (Yes/No)	Ref.
$Cs_3Cu_2I_5/CsCu_2I_3$	(0.32, 0.34)	-	6053	0.70	310	No	[15]
$Cs_2Na_{0.4}Ag_{0.6}In/BiCl_6$: Mn^{2+}	(0.38, 0.42)	82.6	4323	-	310	Yes	[16]
$Cs_3Cu_2I_5/CsCu_2I_3$	(0.32, 0.33)	87.3	-	-	310	No	[17]
$Cs_3Cu_2I_5/CsCu_2I_3$	(0.32, 0.33)	88.4	5877	54.6	310	No	[18]
$CsCu_2I_3$@anthracene	(0.31, 0.31)	83	6718	-	310	No	[19]
$Cs_2K_{0.9}Mn_{0.05}In_{0.9}Sb_{0.1}Cl_6$	-	-	4252	-	310	Yes	[20]
Cs_2ZrCl_6:1%Sb^{3+}	(0.32, 0.38)	-	6020	-	310	Yes	[21]
Cs_2ZrCl_6:10%Sb^{3+}	(0.37, 0.38)	-	4238	-	310	Yes	[21]
$Cs_2NaInCl_6$: Sb^{3+}, Sm^{3+}	(0.30, 0.28)	82	8035	37.5	310	Yes	**This work**

References

[1] P. Han, X. Mao, S. Yang, F. Zhang, B. Yang, D. Wei, W. Deng, K. Han, *Angew. Chem. Int. Ed.* **2019**, *58*, 17231-17235.

[2] I.E. Kolesnikov, E.V. Golyeva, M.A. Kurochkin, E.Y. Kolesnikov, E. Lähderanta, *J. Lumin.* **2020**, *219*,116946.

[3] M.G. Nikolić, D.J. Jovanović, V. Đorđević, Ž. Antić, R.M. Krsmanović, M.D. Dramićanin, *Phys. Scripta* **2012**, *149*, 014063.

[4] A. Zhang, Z. Sun, M. Jia, Z. Fu, B.C. Choi, J.H. Jeong, S.H. Park, *J. Alloy. Compd.* **2021**, *889*, 161671.

[5] L.R. Đačanin, S.R. Lukić-Petrović, D.M. Petrović, M.G. Nikolić, M.D. Dramićanin, *J. Lumin.* **2014**, *151*, 82-87.

[6] M. Back, E. Trave, J. Ueda, S. Tanabe, *Chem. Mater.* **2016**, *28*, 8347.

[7] R. Sun, X. Wei, H. Yu, P. Chen, H. Ni, J. Li, J. Zhou, Q. Zhang, *Dalton Trans.* **2023**, *52*, 2825.

[8] A. Bindhu, A. S. Priya, J. I. Naseemabeevi, S. Ganesanpotti, *J. Alloy. Compd.* **2022**, *893*, 162246.

[9] Z. Zheng, J. Zhang, X. Liu, R. Wei, F. Hu, H. Guo, *Ceram. Int.***2020**, *46*, 6154.

[10] S.-H. Yang, Y.-C. Lee, Y.-C. Hung, *Ceram. Int.***2018**, *44*, 11665.

[11] P. Cai, L. Qin, C. Chen, J. Wang, H. J. Seo, *Dalton Trans.* **2017**, *46*, 14331.

[12]S. Jana, A. Mondal, J. Manam, S. Das, *J. Alloy. Compd.* **2020**, *821*, 153342.

[13]F. Mo, X. Zhang, Z. Sun, Z. Zhu, Z. Guo, Z.-c. Wu, *Ceram. Int.***2019**, *45*, 12319.

[14] J.-Y. Li, D. Hou, Y. Zhang, H. Li, H. Lin, Z. Lin, W. Zhou, R. Huang, *J. Lumin.* **2019**, *213*, 184.

[15] S. Liu, H. Liu, G. Zhou, X. Li, S. Wang, *Chem. Eng. J.***2022**, *427*, 131430.

[16] G. Yang, Y. Zhu, X. Li, J. Huang, X. Xu, X. Ji, A. Wang, J. Cheng, G. Pan, *Opt. Lett.* **2021**, *46*, 6043.

[17] S. Li, F. Weng, P. Yang, X. Li, X. Cheng, L. Zhang, H. Wang, W. Zhang, Z. Zhang, K. Yang, *J. Alloy. Compd.* **2023**, *932*, 167590.

[18] L.-T. Wang, Z.-Z. Ma, F. Zhang, M. Wang, X. Chen, D. Wu, Y.-T. Tian, X.-J. Li, Z.-F. Shi, *J. Mater. Chem. C* **2021**, *9*, 6151.

[19]W. Liu, K. W. Ng, H. Lin, Z. Dai, J. Xu, S. Su, Z. Tang, S. Wang, *J. Phys.Chem.C* **2021**, *125*, 13076.

[20]M. Cong, Q. Zhang, B. Yang, J. Chen, J. Xiao, D. Zheng, T. Zheng, R. Zhang, G. Qing, C. Zhang, K. Han, *Nano Lett.* **2021**, *21*, 8671.

[21] C. Chen, J. Xiang, Y. Chen, M. Jin, J. Zheng, N. Zhang, C. Guo, *Ceram. Int.* **2022**, *48*, 1851.
